# Ooutbreak of livestock-associated methicillin-resistant *Staphylococcus aureus* in a nursing home

**DOI:** 10.1186/1753-6561-5-S6-P168

**Published:** 2011-06-29

**Authors:** E Verkade, T Bosch, Y Hendriks, J Kluytmans

**Affiliations:** 1 Laboratory for Microbiology and Infection Control, Amphia Hospital, Breda, Netherlands; 2 Laboratory for Medical Microbiology and Immunology, Elisabeth Hospital, Tilburg, Netherlands; 3 National Institute of Public Health and the Environment, Bilthoven, Netherlands; 4 Department of Medical Microbiology and Infection Control, VU medical center, Amsterdam, Netherlands

## Introduction / objectives

We describe an outbreak of livestock-associated methicillin-resistant *Staphylococcus aureus* (LA-MRSA) in a nursing home in the Netherlands from October 2010 to February 2011. The nursing home consists of three separate wards and is located in the southeast of the Netherlands, an area with a high pig-density.

## Results

In October 2010, MRSA was cultured from a wound of a resident of the nursing home. Subsequent screening revealed six additional residents and four healthcare workers with MRSA colonization. The colonized residents were located on two wards of the nursing home. The outbreak was controlled by short term isolation of affected residents and decolonization of carriage. Six of the seven affected residents were successfully decolonized. The resident who failed treatment had regularly contact with livestock. Two of the four colonized healthcare workers reported frequent contact with livestock. An epidemiological analysis was performed and the strains were typed using pulsed field gel electrophoresis (PFGE) with *Cfr*9I digestion. PFGE revealed that the MRSA of all residents and the two healthcare workers who did not report contact with livestock were identical. The two healthcare workers who did have contact with livestock had different strains.

## Conclusion

This outbreak shows that LA-MRSA can spread in nursing homes. It can be controlled using relatively simple control measures. PFGE was a useful tool to assist epidemiologists in the control of LA-MRSA.

## Disclosure of interest

None declared.

